# Osteopontin isoform c promotes the survival of cisplatin-treated NSCLC cells involving NFATc2-mediated suppression on calcium-induced ROS levels

**DOI:** 10.1186/s12885-021-08495-z

**Published:** 2021-06-29

**Authors:** Jing Huang, Mu Hu, Huan Niu, Jing Wang, Yang Si, Shan Cheng, Wei Ding

**Affiliations:** 1 School of Basic Medical Sciences, Capital Medical University, Beijing, 100069 China; 2 Department of Thoracic Surgery, Beijing Friendship Hospital, Capital Medical University, Beijing, 100050 China

**Keywords:** Osteopontin, Splicing isoform, NFATc2, Tumor microenvironment, Reactive oxygen species

## Abstract

**Background:**

Tumor microenvironment (TME) critically contributed to the malignant progression of transformed cells and the chemical responses to chemotherapy reagents. Osteopontin (OPN) is a secretory onco-protein with several splicing isoforms, all of which were known to regulate tumor growth and able to alter cell-cell or cell-TME communication, however, the exact role and regulation of the OPN splicing isoforms was not well understood.

**Methods:**

In this study, the effects of conditioned medium from the culture of OPN splicing isoforms overexpressing cells on cell functions were evaluated. The methods of nuclear calcium reporter assays and subcellular distribution of nuclear factor of activated T cells c2 (NFATc2) assays were used to investigate the molecular mechanism underlining the roles of OPN splicing isoforms.

**Results:**

We found that the survival of NSCLC cells treated with cisplatin was increased by secretory OPNc in the condition medium, where reduction of apoptosis by OPNc was associated with the activation of cellular calcium signals and subsequent nuclear translocation of NFATc2.

**Conclusions:**

The results revealed a mechanism of OPN and downstream signal for tumor cells to survive in chemo-stressed TME, which emphasized the importance of secretory proteins in alternative splicing isoforms. Our study not only demonstrated the importance of OPN neutralization for anti-tumor effects, but also implied that modulation in calcium/NFATc2/ROS axis could be a novel approach for improving the long-term outcome of NSCLC treatment.

**Supplementary Information:**

The online version contains supplementary material available at 10.1186/s12885-021-08495-z.

## Background

Platinum-based schemes are a cornerstone for the treatment of non-small cell lung cancer (NSCLC) for broadly used formulas. However, the 5-year survival remained poor for patients receiving cisplatin administration, largely due to the emergence of drug resistance during or even prior to the course of treatment [1]. Chemotherapy resistance, a most fundamental problem in cancer medication, is giving rise to disease progression and tumor recurrence in both local and distant tissues. Tumor microenvironment (TME) is increasingly recognized to affects both the malignant progression and cell responses to chemotherapy agents. Secreted from tumor or stromal cells, various cytokines and growth factors in rich abundance contribute to the aberrant growth, angiogenesis, metastasis and drug resistance. To identify and subsequent deplete the involved key factors is considered as an effective approach to attenuate the development of chemo-resistance of cancer cells.

Osteopontin (OPN) is a member of the small integrin-binding ligand N-linked glycoprotein (SIBLING) protein family. SIBLING family proteins are structurally characterized by their small flexible structure and the presence of an Arg-Gly-Asp (RGD) integrin-binding sequence [2]. OPN is detected in high levels in both plasma and TME in various solid tumor patients, including NSCLC. OPN was previous recognized as a biomaker to associate with cancer progression and drug resistance in NSCLC and other cancer types [3, 4]. As a secreted molecule from a number of cell types is implicated in a variety of biological functions, including cell adhesion, migration, immune response, bone calcification, and tumor progression [5]. NSCLC cell lines of OPN overexpression are found to have more invasive behavior, greater metastatic potential [6] and more resistance to cancer cell drugs [7], nonetheless, the molecular pathways responsible for the oncogenic effects of OPN are still not adequately understood.

In fact, OPN has multiple existing forms from pre- and post-translational modifications. There are three different splicing isoforms (OPN SI) of *opn* gene in human. The dominant OPN protein is OPNa, the full-length form with a molecular weight of ∼54 kD. Two shorter forms are OPNb and OPNc, lacking exon 5 (∼50 kD) and exon 4 (∼47 kD) of the seven OPN exons respectively [8]. In addition to CD44 binding domain and several thrombin or matrix metalloproteinase cleavage sites, human OPN isoforms share a common RGD and SVVYGLR integrin binding sequence [9]. Trans glutamination is thought to alter the conformation of OPN, and thus significantly alter the function of OPN. Transglutaminase 2 catalyzes the polymerization of OPN on multiple Gln residues encoded by exon 2–5. The deleted exons 5 (OPNb) and 4 (OPNc) contain one and three Gln residues, respectively, so that the degree of polymerization of isoforms is proportional to the number of trans glutamination sites, OPNa > OPNb > OPNc. The regulation and exact function of different OPN-SIs are still unclear, but recent reports suggest that some OPN-SIs could be much effective in promoting the development of tumor. Among the known OPN-SIs, OPNc has been revealed to be the most important clinical values. OPNc expression was up-regulated in ovarian, breast and pancreatic cancer, which was related to the effects of OPN-SIs on drug resistance signaling in cellular microenvironments.

It has been reported that OPN interacted with integrin αvβ3 to activate tyrosine kinase Pyk2, c-Src, phosphatidylinositol 3-kinase and phospholipase Cγ in osteoclasts [10]. Activation of phospholipase Cγ (leading to intracellular Ca^2+^ release) or Ca^2+^ influx through channels would lead to the increase of cytosolic free calcium concentration ([Ca^2+^]i) [11]. It was not characterized whether OPN could induce ([Ca^2+^]i) elevation in cancer cells. Upon the increase of [Ca^2+^]i, nuclear factors of activated T cells (NFATc1-c4) are activated by the serine/threonine phosphatase calcineurin, and subsequently translocated to the nucleus to transcribe target genes, such as IL-2, IL-4 and the chemokine CCL21 [12]. NFATs are involved in regulating a variety of cellular functions, including growth, immune response and inflammatory response [13, 14]. Dysregulation of NFAT signaling is associated with malignant phenotypes and progression of tumor [15, 16]. Among various NFATs, NFATc1 (NFAT2) and NFATc2 (NFAT1) seemed to be essential for the regulation of tumor related functions, including cell survival/proliferation, invasive migration and angiogenesis [17, 18].

Currently the role of special OPN isoforms in cancer was obscured, including in the activation of Calcium signaling. From the present investigation, we discovered that supplementing OPN-SIs as in the secretory forms with the conditioned medium altered the cell sensitivity to cisplatin in NSCLC cells. OPNc could promote the cell survival through transmitting stimulatory signal to NSCLC cells in tumor microenvironment (TME), involving the activation of cellular calcium levels and the subsequent translocation of the nuclear factor of activated T cells c2 (NFATc2), eventually suppressing ROS levels and attenuating cell apoptosis. Our findings suggested that OPNc might be a potential factor to transmit environmental signals to cancer cells and confer an adaptive response to pharmacological effects via Ca^2+^/NFATc2/ROS signaling.

## Methods

### Tissue culture and cell treatments

Human embryonic kidney cells (HEK-293 T) were obtained from the American Type Culture Collection (ATCC) (Manassas, VA). Cells were maintained as earlier described [19], at 37 °C in a 5% CO_2_ incubator in Dulbecco’s modified Eagle’s medium (DMEM) with 10% fetal bovine serum (FBS) (Biological Industries, Kibbutz Beit Haemek, Israel) and 1% penicillin-streptomycin (Keygen Biotech, Nanjing, China). The human non-small cell lung cancer cell line H1299 and A549 cells were from the American Type Culture Collection (ATCC). Cells were maintained at 37 °C in a 5% CO_2_ incubator in Ham’s F12 Nutrient Medium (Ham’s F12) with 10% FBS and 1% mixed antibiotics. Cisplatin (QILU Pharmaceutical Co. Ltd., Shandong, China) and FK506 (an inhibitor to calcineurin) (T2144, TagerMol, Target Molecule Corp.) were used in the assays to evaluate cell survival and NFATc2 translocation responsive to nuclear calcium signal.

### Preparation of the conditioned mediums (CMs) containing OPN-SIs

The conditioned mediums were prepared and collected based on the procedure of previous study [20]. Briefly, a number of 2.0 × 10^6^ HEK-293 T cells were seeded in 60 mm plates. After transfected with the plasmids carrying the expression cassette of OPNa, OPNb and OPNc for 6 h, cells were cultured in 4 ml fresh culture medium with FBS for 48 h. The supernatant culture mediums were collected and freeze concentrated to 200 μl using Lyophilizer (Alpha 1-2LDplus, Martin Christ, Osterode am Harz, Lower Saxony, Germany). In assays, 10 μl CM was added to 1 ml fresh medium for conditioned culture.

### Vector preparation and transfection

The expression cassettes of OPN-SIs (OPNa, OPNb and OPNc) were cloned individually into AAV-Ires-hrGFP vectors by NEBuilder HiFi DNA Assembly Cloning Kit (NEB Express Iq). The expression cassettes of NFATs (NFATc1, NFATc2, NFATc3 and NFATc4) were cloned individually into pENTER vectors by Vigene Bioscience Co., Ltd. (Shandong, China) and amplified in bacterial host cells. The constructs of NFATc2M (deletion of 421–428 amino acid residues in the DNA binding domain) [21] and NFATc2MP (mutations in R112A, E114A and T116A, impaired in calcineurin-dependent nuclear import) [22] were generated from the wild type with the use of the Q5® Site-Directed Mutagenesis Kit (E0554S, NEB). The pcDNA3.1-GCaMP6s-tdTomato plasmid was generously transferred from Dr. H Ye [23]. The cells were transfected with plasmids using a Lipofectamine™ 2000 Transfection Kit (Invitrogen, Waltham, Massachusetts, USA) following the vendor’s recommended protocols. Western blotting was performed to detect the protein levels of the corresponded genes at 48 h post transfection.

### Cell viability assay

H1299/A549 Cells were set up at 2× 10^3^ cells per well of a 96-well plate and were cultured in medium overnight. Then the medium was refreshed with medium containing the different treatments according to figure legends. Cell viability was analyzed by Cell Counting Kit 8 (KeyGEN BioTECH, Jiangsu, China) according to the manufacturer’s instructions, based on the procedure of previous studies [20]. The plates were scanned at 450 nm for absorbance using a spectrophotometer (BioTek, Winooski, VT, USA). Each data point was measured for the average from six duplicates. The experiments were repeated independently for 3 times.

### Apoptosis assay

The cell apoptosis was assayed using an Annexin V-FITC Apoptosis Detection Kit (KeyGEN BioTECH, Jiangsu, China) following the manufacturer’s standard protocol, based on the procedure of previous studies [20]. Briefly, cells were seeded in 6-well plates at 2.0 × 10^5^ cells/well and treated with cisplatin at 30 μg/ml for 48 h or 20 μg/ml for 48 h, except the control group where DMSO was applied as the vehicle used to dilute drugs.

### Cellular calcium imaging

Cellular calcium analyses were performed as earlier described [20]. Cells cultured on coverglass were transfected with pcDNA3.1-GCaMP6s-tdTomato. Live confocal microscopy was performed at 24 h post transfection using an UltraVIEWVoX system (PerkinElmer, Waltham, MA, USA). Fluorescent signals from two fluorophores (GCaMP6s and tdTomato) were collected at 488 nm/543 nm for excitation and 493–552 nm/560–605 nm for emission. Images were acquired by scanning in the frame scan mode (512 pixels, 1 s/frame). The fluorescence intensity and the ratio of GCaMP6s to tdTomato channels (expressed as G/R) were calculated and plotted using a custom macro script for Image J 1.4 (National Institutes of Health, Bethesda, MD, USA).

### Immunofluorescence

The method has been widely implemented by our laboratories [24]. The cells on coverslips were probed with Anti-NFATc1 (MA3–024, Thermofisher), Anti-NFATc2 (JA11–08, HuaAn Biotechnology Co., Ltd., Hangzhou, China), Anti-NFATc3 (sc-8405, Santa cruz), and Anti-NFATc4 (YT3085, Immunoway, Suzhou, China) primary antibody. After the incubation with an Alexa Fluor® 488 (for primary antibody of anti-NFATc1 and anti-NFATc3) (Life Technologies, Massachusetts, USA) for and an Alexa Fluor® 594 secondary antibody (for primary antibody of anti-NFATc2 and anti-NFATc4) (Life Technologies, Massachusetts, USA), the cells were stained with Hoechst 33258 (Sigma-Aldrich, St. Louis, MO, USA) for nucleus label. Finally, the cells were visualized with a confocal microscope (Leica Microsystems TCS SP8. Wetzlar, Germany). Control samples without adding the primary antibody were prepared for determining the level of non-specific noise.

### Cytoplasmic and nuclear extracts

Nuclear and cytoplasmic extracts of cells were prepared using nuclear and cytosol extraction kit from Applygen, as earlier described [25]. Whole-cell extracts were prepared by lysing the cells in ice-cold CEB-A buffer (Applygen Technologies Inc., Beijing, China). The lysates were then solubilized via vortex for 30 s and placed on ice for 10 min. The resulting homogenate was centrifuged for 5 min (12,000 g) at 4 °C and the supernatant was kept as the cytoplasmic fraction. The precipitate was resuspended in 40 μl nuclear lysis buffer. The lysates were placed on ice for 30 min and solubilized via vortex for 15 s every 10 min. The resulting homogenate was centrifuged for 5 min (12,000 g) at 4 °C and the supernatant was kept as the nuclear fraction. Samples were stored at − 80 °C.

### Western blotting

Western blot analyses were performed as earlier described [6]. Briefly, samples of cell lysates were prepared and separated by 10% SDS-polyacrylamide gel electrophoresis (SDS-PAGE), then transferred onto polyvinylidene fluoride (PVDF) filters. The probing antibodies were against the following antigens: OPN (ab8448, abcam), NFATc2 (JA11–08, HuaAn Biotechnology Co., Ltd., Hangzhou, China), NFATc3 (sc-8405, Santa cruz), FLAG (#14793, CST) and GAPDH (TA-08, ZSGB-BIO, Beijing, China).

### mRNA-sequencing and data processing

Cells were scraped off from the surface in trypsin-versene solution and collected by 500 g centrifugation. The pellet was washed with PBS to remove residual media. Total RNA extractions were performed with the RNA-Quick Purification Kit (ES-RN001, Yishan Biotechnology Co., Ltd., Shanghai, China) following the manufacturer’s protocol. The RNA concentrations were determined using a Nanodrop ND1000 spectrophotometer (Thermo Scientific). The mRNA sequencing libraries were performed with the VAHTS Universal V6 RNA-seq Library Prep Kit for Illumina (NR604–01, Vazyme Biotech, Nanjing, China), VAHTS RNA Multiplex Oligos Set1- Set2 for Illumina (N323, Vazyme Biotech, Nanjing, China), VAHTS DNA Clean Beads (N311–01, Vazyme Biotech, Nanjing, China), VAHTS mRNA Capture Beads (N401–01, Vazyme Biotech, Nanjing, China). The quality assessments were performed in the laboratory of GENEWIZ, lnc (Suzhou, China). All prepared samples subjected to paired-end multiplex sequenced (2 × 150 bp) on the Illumina Hiseq X10 platform. Approximately 8 Gb sequencing data was generated for each sample.

The clean reads in compressed FASTQ format were aligned using HISAT2 to the reference of human genome (Homo_sapiens.GRCh38.dna.primary_assembly.fa, European Molecular Biology Laboratory (EMBL)) with matched rates over 90%. The resulted BAM files were sorted with SAMtools (version 1.18). The depth counts were called with HTSeq (version 0.11.2. Linux_x86_64, Simon Anders (sanders@fs.tum.de)) with the reference of human genome (Homo_sapiens.GRCh38.94.gtf) used to calculate the Fold change (FC) of FPKM and *p* value among sample groups according to an over-dispersed Poisson model. The *p* value of genes was calculated using DESeq2 (version 1.30.0). Differentially expressed (DE) genes were identified with the thresholds of both expression counts≥1.2 and expression counts≤0.75 in mean expression and FDR ≤ 5% using Benjamin-Hochberg procedure. The enrichment of DE genes was performed using the Gene Ontology Tools (https://go.princeton.edu/) and Cluster 3.0 (Michael Eisen, Stanford University), then displayed with TreeView (version 1.1.6r4, Alok Saldanha).

### RNA extraction and qRT-PCR

Total RNA was isolated using RNA-Quick Purification Kit (Yishan Biotechnology Co., Ltd., Shanghai, China). HiScript II Q RT SuperMix Kit (Vazyme, Nanjing, China) was used for reverse transcription. ChamQ SYBR qPCR Master Mix (Vazyme, Nanjing, China) was used to quantify gene expression level from the obtained cDNA. The qRT-PCR amplified samples were performed using Archimed X6 (Rocgene, Beijing, China). The primers for detecting NFATc2, CYP2E1, CYBB, RAC2, ATP6V0C, TRADD, MAFK, MAFG, ATP6V0D2, ALCAM, ATP6V1H, GPX8 and SOX2 listed in Table S1. GAPDH was used as the loading reference.

### Measurement of ROS level

The fluorescent probe H2DCFDA was used to measure the intracellular generation of ROS. Briefly, cells were plated in 12-well culture plates, incubated overnight, and treated with cisplatin for 12 h. The cells were incubated at 37 °C with 5 μM H2DCFDA (Beyotime, Shanghai, China) for 20 min. Fluorescence intensity was measured by EPICS@X flow cytometer (Beckman Coulter, USA) with extinction and emission at 488 and 525 nm, respectively, and data then were evaluated with FlowJo VX 14.0 software.

### Statistical analysis

The analysis of variance (ANOVA) was used to determine the statistical significance of data in multiple groups. The Student’s t-test was used to compare cell functions between paired groups. Cases of *p*-value< 0.05 was defined as statistically significant. The program of Prism 8 (GraphPad Software, Inc., La Jolla, CA, USA) was used for data plotting.

## Results

### Culture with conditioned medium from OPNc overexpressed cells decreased the sensitivity to cisplatin in H1299 cells

**Fig. 1:**
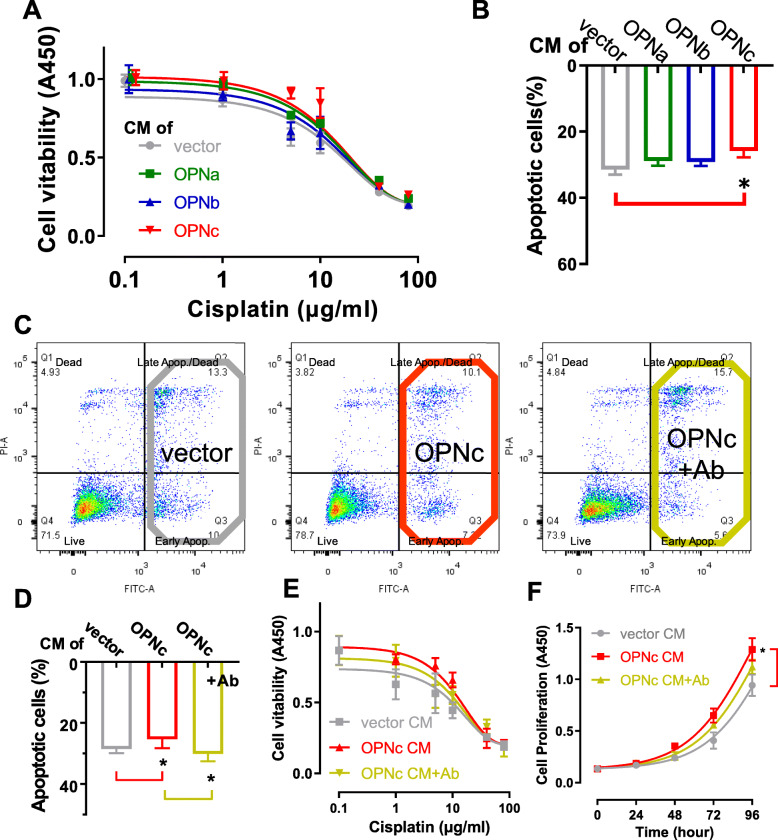
Decreased sensitivity to cisplatin observed in OPNc conditioned cultures of NSCLC cells. **a** Survival of H1299 cells cultured in OPN-SI CMs in the dose-response of cisplatin by CCK8 assays. **b** Cell apoptosis quantified from H1299 cells cultured in OPN-CMs and treated with cisplatin 30 μg/ml for 48 h. **c** Effect of OPNc on apoptosis as shown from the sample results of flow cytometry. **d** Quantification of cell apoptosis in H1299 cells treated with cisplatin 30 μg/ml for 48 h cultured in OPNc-CM with or without 1 μl OPN-neutralizing antibody added to 1 ml fresh medium for conditioned culture. **e** Cell survival from CCK8 assays in H1299 cells subjected to cisplatin doses in OPNc-CM and CM with OPN-neutralizing antibody. **f** Cell growth in H1299 cells in OPNc-CM and neutralizing antibody. Data were represented as the mean ± SD from 3 (apoptosis) or 6 (viability) independent experiments. * *p* < 0.05

Secreted OPN proteins were previously reported to influence the sensitivity of certain anti-cancer drugs in hepatocellular carcinoma, colon cancer and glioma cells [20, 26]. In NSCLC cells, to further investigate whether such effect is rendered by specific OPN-SIs, we prepared conditioned medium (CM) by transfecting different OPN-SIs into HEK-293 T cells (Fig. S1A, B). When H1299 cells were cultured in the obtained CMs (Fig. 1A), we found the OPNc-CM exerted a stronger effect to reduce cisplatin sensitivity than OPNa-CM and OPNb-CM. From apoptosis assays, CMs of OPNa, OPNb or OPNc reduced the sensitivity of H1299 cells to cisplatin following 30 μg/ml cisplatin treatments for 48 h, from 31.4% to 28.8%, 29.1% and 25.8%, respectively (Fig. 1B), consistently with the OPNc group to be the most significant. To verify if OPNc was indeed a casual factor in the CM, we used an OPN antibody for targeted depletion before applying the CM of cell cultures. We found that the OPN effects on drug sensitivity (Fig. 1C-F) could be neutralized. These data indicated that the secretory OPNc served as the most potent isoform to alter the sensitivity of cisplatin in H1299 cells and increased the survival from apoptosis.

### OPNc increased intracellular calcium level and activate calcium signal in H1299 cells

**Fig. 2:**
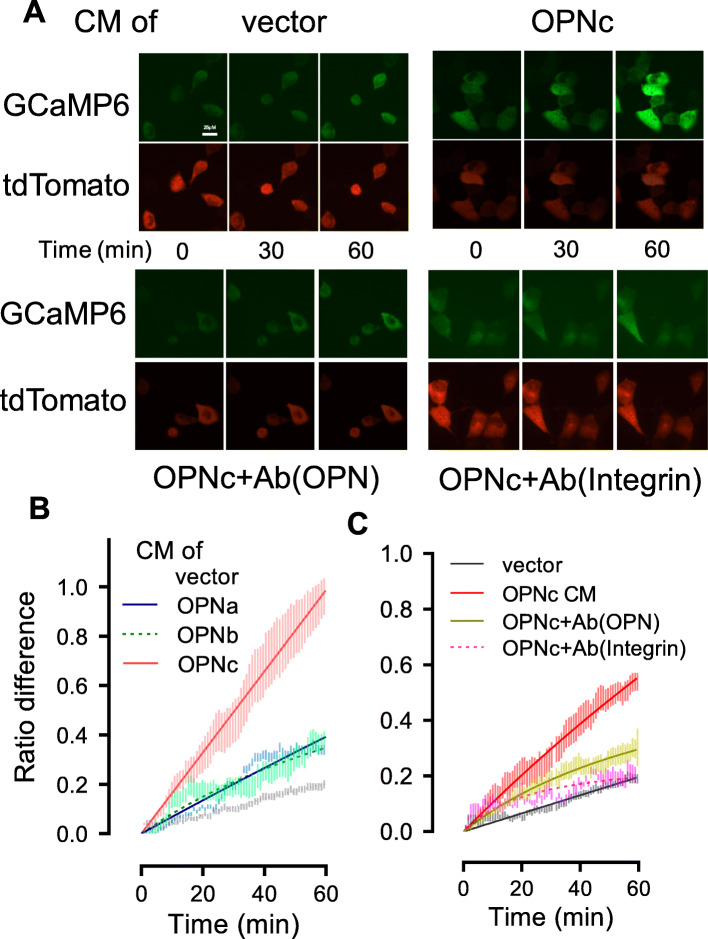
Addition of conditioned medium from OPNc overexpressed cells increased intracellular calcium levels in H1299 cultures. **a** Live fluorescence images for [Ca^2+^]i in H1299 cells transfected with GCaMP6s-tdTomato plasmid and stimulated with OPNc-CM, CM with addition of OPN-neutralizing antibody and integrin αvβ3-blocking antibody (ab78289). **b** Quantitative analyses for [Ca^2+^]i in H1299 cells transfected with GCaMP6s-tdTomato and stimulated with OPN-CMs. **c** Quantitative analyses for [Ca^2+^]i in H1299 cells stimulated with OPNc-CM, CM with addition of OPN-neutralizing antibody and integrin αvβ3-blocking antibody. A total of 10 to 30 individual cells were analyzed for ratios of GCaMP6s over tdTomato fluorescence intensities. Data were represented as the mean ± SD

OPN was demonstrated in osteoclasts to interact with integrin αvβ3 and elevated [Ca^2+^]i [27]. Using a GCaMP based reporter, we detected the changes in cytosolic calcium concentration in transfected H1299 cells following the treatments with OPN-CMs. As shown in Fig. 2A, the CM of OPNc was able to elicit a fast and robust stimulation in [Ca^2+^]i, much more intensive than cases of OPNa and OPNb (Fig. 2B). When the OPNc-CM was neutralized by antibody, the calcium signal was significantly reduced (Fig. 2A, C). The addition of the antibody to integrin αvβ3 also significantly abolished the OPNc-CM induced the increase of [Ca^2+^]i (Fig. 2A, C). This suggested that OPNc could be a potent factor in the secretory environment to trigger the activation of cellular calcium signals.

### OPNc-CM induced the nuclear localization of NFATc2 in H1299 cells

**Fig. 3:**
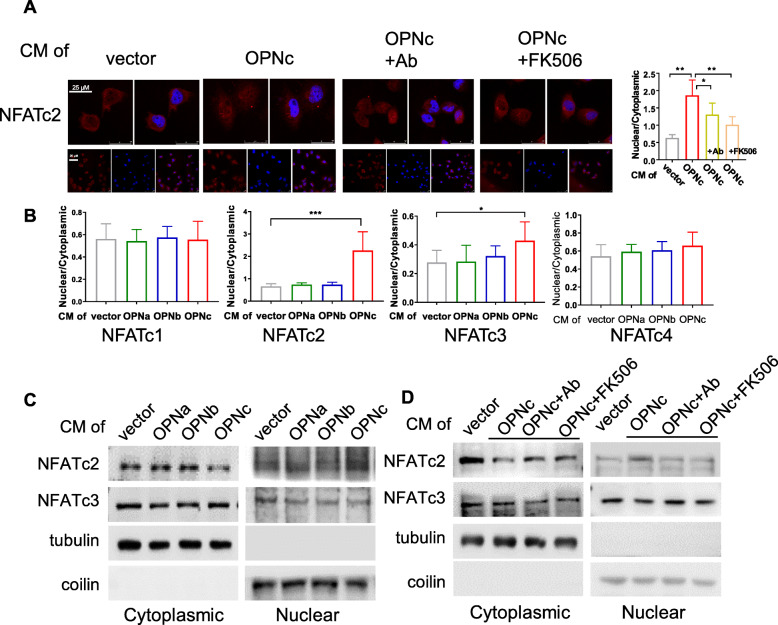
Nuclear accumulation of NFATc2 in cells treated with OPN-CMs. **a** Immunofluorescent images for NFATc2 subcellular localization in cells induced by OPNc-CM and CM with addition of OPN-neutralizing antibody or FK506 as an inhibitor to calcineurin. Twenty individual cells were selected for quantitative analyses of the ratio between nuclear localization and cytoplasmic localization for each NFATc2 as shown in the plot. **b** Quantification of NFATc1-c4 nuclear localization from immunofluorescence in cells treated with OPN-CMs for 3 h. **c** Western blot analyses for cytoplasm or nuclear distribution of NFATc2 and NFATc3 proteins in cells treated with OPN-CMs, where tubulin and coilin were used as cytoplasm and nuclear makers respectively. **d** Western blot analyses for cytoplasm or nuclear distribution of NFATc2 and NFATc3 proteins in cells treated with OPNc-CM and CM with addition of OPN-neutralizing antibody or FK506. * *p* < 0.05, ** *p* < 0.01, *** *p* < 0.001

An important known transcription factor responded to calcium signals is NFAT, which can be activated by calcineurin as a Ca^2+^/calmodulin-dependent protein phosphatase. Reports have shown that NFAT signaling was responsible for cell survival and resistance from cytotoxicity in various cancer cells [28–30]. Several different forms of NFAT can be found in NSCLC cells. We used immunofluorescent assays to examine the types and distribution of major NFAT species. OPNc-CM treatments triggered significant nuclear translocation of NFATc2 at 3 h (Fig. 3A), which could be blocked by OPN antibody or pretreatment of FK506, a blocker of calcineurin activity. The nuclear translocation of NFATc3 was also observed in the cells exposed to OPNc-CM in much small amounts, but not seen in NFATc1 and c4. Comparing to OPNc. OPNa-CM and OPNb-CM were absent for the abilities to promote the nuclear translocation of any of NFATc1-c4 in H1299 cells (Fig. 3B). From the western blot probing for NFATc2 or NFATc3 in both cytoplasmic and nuclear fractions, the effect of OPNc-CM to increase nuclear translocation of NFATc2 was verified (Fig. 3C), but not for NFATc3, which was much less significant. In antibody neutralization and FK506 treatment samples, reduced nuclear accumulation of NFATc2 was detected by the western blot assays (Fig. 3D). These results demonstrated that NFATc2 was the primary nuclear transported signal molecule to selectively mediate the stimulation of OPNc, and possibly drive the transcription of specific gene to promote the growth and survival of cancer cells.

### Transcriptome screening revealed genes involved in ROS metabolism were enriched downstream of OPNc/NFATc2 signaling

**Fig. 4:**
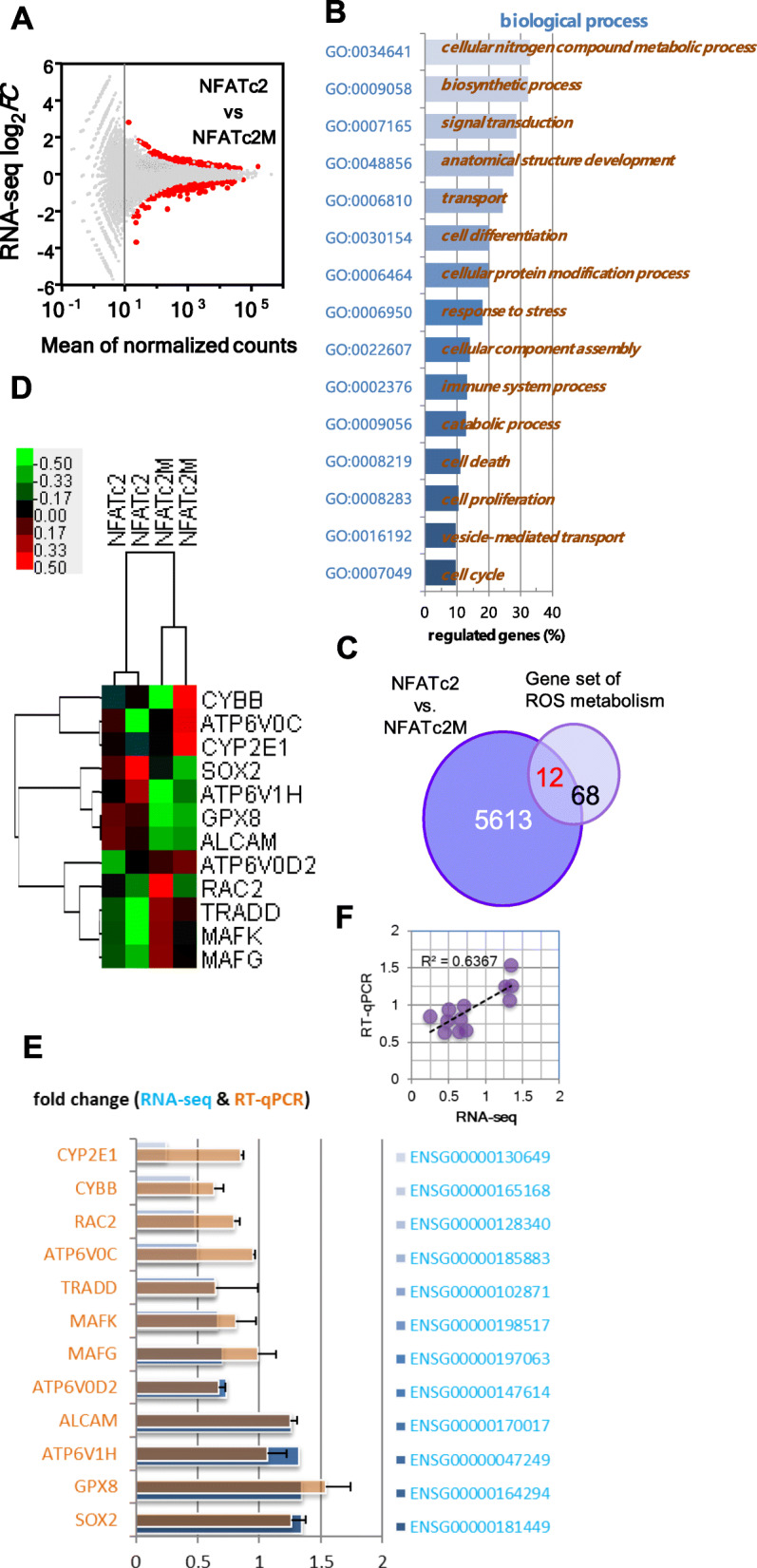
Transcriptome analysis of A549 cells transfected with NFATc2 or NFATc2M (loss of function mutant) and stimulated by OPNc-CM. **a** MA plot shows the log2-fold change (FC) value attributable to a given gene over the mean of normalized counts. Genes with the adjusted *p* value < 0.01 were highlighted in red. **b** Enriched Gene Ontology sets for differentially expressed (DE) genes. **c** Venn diagram illustrating the number of genes between DE genes with a most enriched gene set of ROS metabolism. **d** Heatmap representing the identified 12 DE genes of detected expression levels in hierarchical clusters. **e** Validation of the mRNA levels of selected DE genes by qRT-PCR (mean ± SEM. *n* = 3) and compared with RNA-seq counts. **f** Correlational analysis on RNA-seq and qRT-PCR results

Took the advantage from earlier findings in NFAT studies, we deployed a mutant NFATc2 – NFATc2M with the deletion in the DNA binding domain, as a loss of function reference to search for OPNc responsive genes that are regulated by wild type NFATc2 at transcription levels. A549 cells were transfected with plasmid of NFATc2 or NFATc2M and induced with OPNc-CM. RNA-seq and differential expression (DE) gene analyses were performed. The MA plot to indicated log2-fold changes (FC) attributable to a given variable over the normalized mean in counts for all samples was shown in Fig. 4A. We imposed a threshold of 1.2-fold change (|log2FC| ≥ 0.26) and identified 5625 genes as differentially expressed which qualified from multiple testing (FDR ≤ 5%). The enriched top-level gene ontology categories included the biological processes of cell differentiation, cell proliferation, cell death and cell cycle, suggesting that the important roles in cancer cell survival (Fig. 4B).

Interestingly, when we went through and tried to summarize DE genes in crossover categories, we realized that many of the profiled genes associated with the function of ROS metabolism. Given the fact that multiple earlier report in both OPN and NFAT studies suggested a connection with drug related redox stress in cancers, we extracted 80 genes from Gene Set Enrichment Analysis (GSEA) panels (Table S2) and found that 12 genes were identified as positively identified as DE with statistical significance between NFATc2 and NFATc2M samples (Fig. 4C). The clustered FC heatmap from normalized RNA-seq counts of the identified genes were shown in Fig. 4D. Although there seemed to be a variation between samples with a same treatment group, the RT-qPCR validation gave a very consistent result (Fig. 4E) for their up/down regulated levels and with a good correlation of both assays (Fig. 4F) of RNA-seq and qRT-PCR.

### Addition of OPNc to cultural microenvironment reduced cisplatin induced cellular ROS levels in H1299 cell

**Fig. 5:**
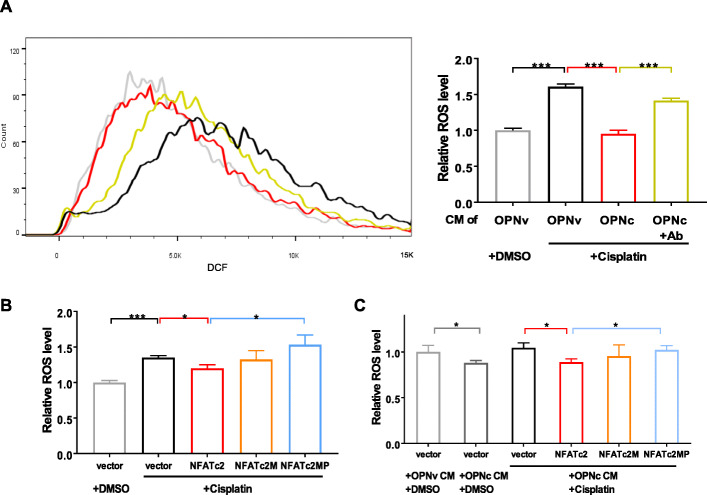
Effects of OPNc on ROS levels in cisplatin treated H1299 cells by DCF fluorescent assays. **a** Flow cytometry from H1299 treated with 20 μg/ml cisplatin in OPN-c (with or without neutralizing antibody) CMs for 12 h. Quantification of the fluorescent intensities was shown as in the plot. **b** ROS levels in H1299 cells transfected with NFATc2 or mutants in 20 μg/ml cisplatin for 12 h. **c** Effect of OPNc-CM on ROS levels in NFATc2 transfected H1299 cells. Data were represented as the mean ± SD from 3 independent experiments. * *p* < 0.05, ** *p* < 0.01, *** *p* < 0.001

To obtain direct evidence of OPNc indeed was able to alter NFATc2-mediated ROS production or clearance, we performed a quantitative fluorescent flow cytometry assay to measure the ROS levels in cisplatin treated H1299 cells. The result in Fig. 5A showed that cisplatin could significantly raise the ROS levels in H1299 cells, where OPNc-CM treatments compromised the increase by approximately 59%. The effect was suggested causual and specific as from the antibody neutralization results (Fig. 5A). To examine whether NFATc2 was involved as mediator molecule, we introduced NFATc2M mutant with FLAG tags into H1299 cells (Fig. S2), as well as a mutant NFATc2MP, impaired in calcineurin-dependent nuclear import with mutations in the dephosphorylation catalytic sites. The results in Fig. 5B showed that the overexpression of wild typed NFATc2 could be helpful to repress cisplatin-induced ROS accumulation, where neither of NFATc2M or NFAT2cMP had a similar function. OPNc-CM most significantly reduced the ROS levels in NFATc2 overexpressed H1299 cells, comparing to other groups (Fig. 5C). These results suggested the importance of a NFATc2 dependent cell protection mechanism for attenuating ROS induced apoptosis in NSCLC cells.

### The role of Ca^2+^/NFATc2 signal on ROS levels in the pro-survival function of OPNc-CM in cisplatin treated A549 cells

**Fig. 6:**
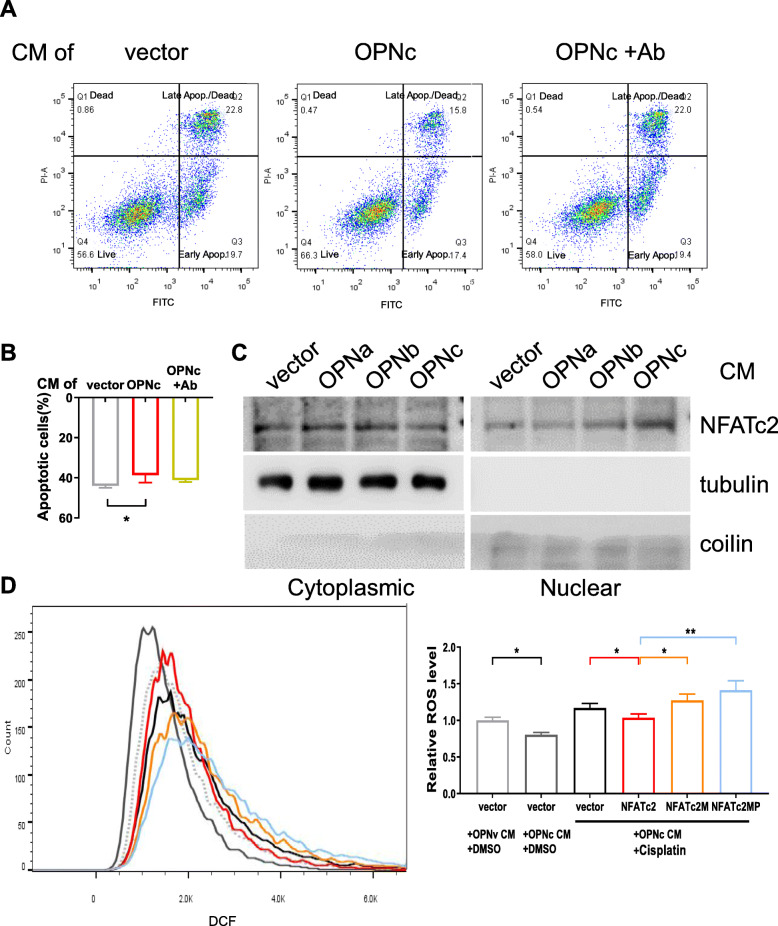
NFATc2 regulated ROS metabolism involved in OPNc-CM promoted cell survival of A549 cells with cisplatin treatment. **a** Apoptosis analyses by flow cytometry in A549 cells treated with cisplatin (20 μg/ml) for 48 h in OPNc-CM and CM with OPN-neutralizing antibody. **b** Quantification of apoptosis from conditions as in a, *n* = 3. **c** Western blot analyses for cytoplasmic or nuclear distribution of NFATc2 in A549 cells treated with OPN-CMs. **d** ROS cytometry in the NFATc2 or mutants transfected A549 cells treated with 10 μg/ml cisplatin in OPNc-CM for 12 h. Data for quantification were represented as the mean ± SD from 3 (for apoptosis and ROS detections) independent experiments. * *p* < 0.05, ** *p* < 0.01

Using A549 as an alternative NSCLC cell model, we verify our previous findings with comprehensive assays focusing on OPNc with the comparison to other OPN-SIs. OPNc-CM reduced the rate of apoptosis from 20 μg/ml cisplatin treatments at 44% to 38.7%, and the effect could be neutralized by the OPN antibody (Fig. 6A, B). The western blot probing for NFATc2 in both cytoplasmic and nuclear forms verified that OPNc-CM increased NFATc2 nuclear accumulation under the experimental conditions (Fig. 6C). We then evaluated the role of OPNc-CM in ROS levels in A549 cells transfected with NFATc2 or its mutants, following the treatment with 10 μg/ml cisplatin for 12 h. OPNc-CM significantly reduced the level of ROS to approximately 88% in NFATc2 overexpressed A549 cells, compared to cell with vector control (Fig. 6D). These results were consistent with our observation in H1299 cells, and therefore indicated that the increase of OPNc-CM induced cell growth in CCK-8 assays and cell survival from cisplatin treatment involved enhanced NFATc2 nuclear translocation, which altered ROS metabolism to reduced cellular ROS levels and ROS induced apoptotic effects.

## Discussion

The resistance to anticancer drugs is a major obstacle in the clinical practice of tumor treatment. Recent studies have shown that molecules secreted by cancer cells are involved in conferring chemotherapy resistance through altering TME [31]. The molecules could be secreted by tumor cells with acquired growth potential and propagated as important signals to synchronize adjacent cells and make the community respond to environmental stress. Several studies have demonstrated growth-promoting effects of tumor cell–derived OPN on adjacent cells in the tumor microenvironment [20, 32]. In addition to its secretion by tumor cell, OPN is also expressed by a variety of cells in the tumor microenvironment, such as macrophages, natural killer cells, and endothelial cells [33]. However, the autocrine and paracrine crosstalk between secreted OPN and various cell types remains unresolved, and the generation and function of splicing isoforms are even less clear. As a minor splicing isoform of OPN, OPNc seems to play an important role in promoting cell survival under drug-induced cytotoxic pressure (Fig. 1). OPNc might be a potential factor to transmit a stimulation signal to adjacent cancer cells and confer an adaptive response to pharmacological insults via Ca^2+^/NFATc2/ROS signaling. Among these secretory factors, splicing isoforms are good candidates. These can be generated and accumulated rapidly from the existing precursor mRNA to achieve the abundance of mediating and transmitting response signals in TME. We believe that alternative splicing isoforms, especially secretory proteins, could provide a wealth of candidates as effective indicators of chemotherapy resistance and potential targets for improving chemotherapy.

In most cases, due to the complexity of TME, it is very challenging to accurately monitor and profile their changing components. Alternatively, analyses can be used to track cellular signaling molecules and downstream effects associated with environmental stimulation, especially to determine the dynamics of rapid responses. As the second messenger of cells, calcium is an important molecule that responds to environmental stimulation and is also related to the molecular function of OPN [34]. It has been reported that the application of soluble OPN or RGD peptides induced the transient elevation of [Ca^2+^]i in human osteoclast-like cells isolated from giant cell tumor of bone [35]. Our previous studies also showed robust responses of nuclear calcium increase in colon cancer cells, when cells were treated with the OPNc-CM [20]. In this study, we observed an increase of [Ca^2+^]i when cells were treated with the OPN-CMs, especially OPNc-CM (Fig. 2). The [Ca^2+^]i could be used as a rapid and sensitive indicator to reflect the response of cells to environmental stimulation in TME. As a microenvironment factor, secreted proteins (such as OPNc) could easily transmit environmental stimulation signals to cells, and also could remodel the cells intracellular phenotypes for the adaptation to TME.

Although we do not know yet how might OPN stimulate the increase of [Ca^2+^]i in NSCLC cells, the remarkable reduction of calcium signal by OPN antibody suggested that such effect was indeed rendered by the OPN protein in the CM (Fig. 2A, C). This response was further inhibited by an antibody directed against the integrin αvβ3 (Fig. 2A, C), suggesting the importance of binding to RGD receptors in inducing transient elevation of [Ca^2+^]i. Ligation of integrin αvβ3 might lead to the phosphorylation and activation of phospholipase C, which could lead to Ca^2+^ release from intracellular stores. The deleted exon 4 of OPNc led to the minor degree of isoform polymerization, comparing with OPNa and OPNb, which could modify protein function by altering the conformational state of OPN, thereby significantly altering OPN function. The precise mechanism underlying OPN-SI-regulated calcium signal in cells is currently under investigation in our laboratory.

In many cell types, activation of Ca^2+^ signaling regulates numerous functions, including secretion and gene transcription. In cancer cells, it has been suggested that the activation of Ca^2+^ signaling is required for activation of NFATs, which is in turn necessary for cell survival. NFAT signaling pathway regulates various aspects of tumor cell functions. As a calcium sensor, NFAT integrates calcium signal with other pathways involved in development and growth, immune response and inflammatory response [36]. Different NFAT members have different functions in different cancers. The exact function of NFAT in tumor or tumor microenvironment depends on cellular conditions. In the present study, NFATc2 was selectively promoted the nuclear translocation by secretory OPNc in NSCLC cells (Fig. 3), may play different functions compared with other members of NFAT family.

Adaptive antioxidant response to alleviate oxidative stress from ROS surge during systemic therapy is one of the most important mechanisms of drug resistance [37]. The cytotoxic effect of cisplatin is mainly mediated by the generation of nuclear DNA adducts. If it cannot be repaired, it will lead to cell death as a consequence of DNA replication and transcription blockage. However, the ability of cisplatin to induce nuclear DNA damage is not sufficient to explain its high efficiency, nor its toxic effects on normal post mitotic tissues. Oxidative damage following cisplatin exposure was observed in several tissues in vivo, suggesting a role of oxidative stress in the pathogenesis of cisplatin induced dose limiting toxicity. It has been reported that cisplatin exposure induces a mitochondrial dependent ROS response. Overproduction of ROS can induce oxidative stress leading to cell death that significantly enhances the cytotoxic effect caused by nuclear DNA damage [38]. Mechanistic investigations identified through the NFATc2/SOX2/ALDH1A1 axis, oxidative stress induced by cancer drug treatment is attenuated, leading to increased resistance in a mutation-independent manner [18]. Indeed, we have observed a set of DE genes clustered in ROS signaling in NFATc2 transfected A549 cells induced by OPNc-CM (Fig. 4), including SOX2, RAC2 and GPX8. SOX2 as a critical transcriptional regulator is amplified in various cancer types and affects cancer cell physiology via involvement in complicated cell signaling and protein-protein interactions, including triggering cell apoptosis by the combined effects of ROS overproduction. Up-regulation of SOX2 in the OPNc-CM induced A549 cells overexpressed with NFATc2 in our study suggests that ROS generation may be compromised. Glutathione peroxidase (GPX) is the most important antioxidant enzyme for maintaining ROS homeostasis, including 8 glutathione peroxidases (*Gpx1–Gpx8*) so far identified [39]. In OPNc-CM induced NFATc2 overexpressed cells, the expression of *Gpx8* was up-regulated. These results suggested that up-regulation of SOX2 and GPX8 could be involved in OPNc/NFATc2 signaling, which could contribute to ROS homeostasis in NSCLC cells. Indeed, increases in ROS induced by the cisplatin treatment were significantly reduced by NFATc2 up-regulation or OPNc-CM induction (Fig. 5).

NFATc2 was up-regulated and ROS remained at a low level compared with parental cells in multiple cell lines with induced resistance to chemotherapy or targeted therapy, suggesting that NFATc2 played an important role in drug resistance by inhibiting ROS level. Recently, a similar mechanism has been reported, involving another NFAT family protein NFATc1. NFATc1, as a coactivator and transcriptional regulator of SOX2, promotes tumor dedifferentiation and epithelial mesenchymal transition (EMT) gene expression in p53 disrupted cells. Literature has shown that NFATc1 activation may induce EMT through SOX2 in an appropriate genetic background [40]. As a specific splicing isoform of OPN, OPNc could be upregulated in response to inflammatory or carcinogenic stimulation and secreted by multiple types of cells in TME. Both in p53 wild-type and mutant NSCLC cells, OPNc as a secretory factor could induce the nuclear accumulation of NFATc2 rather than other NFAT members. These results suggested that the regulatory roles of NFATc2 on the growth and survival of NSCLC cells were not only influenced by its protein expression, but also by environmental stimulation. It suggested OPNc/NFATc2 could rapidly receive and transmit extracellular stimuli, and induce adaptive response of cells to cytotoxic pressure.

## Conclusion

Our study reports a novel NSCLC drug sensitivity pathway which links micro-environmental stimulation to induction of adaptive response of cells, evasion of cell death and enhancement of drug resistance. OPNc could be a potential factor to transmit environmental signals and remodel the cellular endophenotypes for adapting to adverse environment. NFATc2 could be an important target for the treatment of NSCLC, especially for the patients resistant to conventional chemotherapy. In the long run, the combination of classic cancer treatments and new treatment strategies for Ca^2+^/NFATc2/ROS axis could make the treatment of NSCLC patients more effective.

## Supplementary Information

**Additional file 1.**

## Data Availability

Raw data of RNA-seq were available at GEO database repository; accession ID: GSE166966 (all data), GSM5089957 (NFATc2 rep1), GSM5089958 (NFATc2 rep2), GSM5089959 (NFATc2M rep1) and GSM5089960 (NFATc2M rep2).

## Abbreviations

OPN: Osteopontin
NSCLC: Non-small cell lung cancer
TME: Tumor microenvironment
NFATc2: Nuclear factor of activated T cells 2
