# Supplementary material for: Osteopontin isoform c promotes the survival of cisplatin-treated NSCLC cells involving NFATc2-mediated suppression on calcium-induced ROS levels
Source: BMC Cancer. 2021 Jun 29;21:750. doi: 10.1186/s12885-021-08495-z (PMC8243455; doi:10.1186/s12885-021-08495-z)
Supplement: Supplementary file 1 — Additional file 1. [file 12885_2021_8495_MOESM1_ESM.pdf]

Suppl Table 1 The qPCR primers used in this study

Target	Oligonucleotide sequence
CYP2E1	F: GAAGCAACCCGAGACACCAT<br>R: CACACTCGTTTTCCTGTGGA
CYBB	F: AAGTGCCCAAAGGTGTCCAA<br>R: CCCATCAACCGCTATCTTAGGT
RAC2	F: TGGCCAAGGAGATTGACTCGG<br>R: GCCTCGTCGAACACGGTTT
ATP6V0C	F: CCAACGCTGCGGAGATCCA<br>R: CCCATGACGGCGAAAAACG
TRADD	F: CGGAGTAGAGCGGAGCCT<br>R: CTGCCATCTCACCTCCTGGC
MAFK	F: TTCCAGGGAGCTCTGTCCT<br>R: CCCGCCTCCTTCTTGACCTTT
MAFG	F: GCCTCCTATCAGAGAGCGCC<br>R: GATCTCCTCCTTGGACAGGC
ATP6V0D2	F: TCGAAACGCCATTAGCTCCAT<br>R: TGTCGGCCTCAAACTCAAGA
ALCAM	F: ACTTGACGTACCTCAGAATCTCA<br>R: CATCGTCGTACTGCACACTTT
ATP6V1H	F: CCGAGGATTCGCGAGATTTG<br>R: CACGAACTTCTGCAGCCTTG
GPX8	F: CCCAGAGCAAAGGTATTTGCAG<br>R: TTTACAACTAGTGAAACTTTGCCTT
SOX2	F: GCCGAGTGGAAACTTTTGTCG<br>R: GGCAGCGTGTACTTATCCTTCT
NFATc2	F: AGAATCCATCCTGCTGGTTC<br>R: TCCATGTAGCCATGGAGCTG
GAPDH	F: GGAGCGAGATCCCTCCAAAAT<br>R: GGCTGTTGTCATACTTCTCATGG

Suppl Table 2 The constructed gene set of ROS metabolism

Genes	Reference
GCLC, GCLM, GGT1, GPX1, GPX2, GPX3, GSR, GSTA2, GSTA3, GSTA4, GSTM1, GSTM2, GSTM3, GSTM4, GSTM5, GSTP1, GSTT1, GSTZ1, IDH1, MGST2, MGST3, NCF1C	GSEA (ROS metabolism)
ATOX1, CAT, CDO1, DUSP1, GOT1, GPX1, GPX2, GPX3, GPX4, MPO, NUDT1, PDLIM1, PRDX6, SDS, SELENOP, SOD1	GSEA (ROS (glutathione, ox stress))
ATP6V0A1, ATP6V0A2, ATP6V0A4, ATP6V0B, ATP6V0C, ATP6V0D1, ATP6V0D2, ATP6V0E1, ATP6V0E2, ATP6V1A, ATP6V1B1, ATP6V1B2, ATP6V1C1, ATP6V1C2, ATP6V1D, ATP6V1E1, ATP6V1E2, ATP6V1F, ATP6V1G1, ATP6V1G2, ATP6V1G3, ATP6V1H, CYBA, CYBB, HVCN1, LPO, MPO, NCF1, NCF2, NCF4, NOS1, NOS2, NOS3, RAC2, SLC11A1, TCIRG1	GSEA (ROS and RNS production in phagocytes)
GPX8	Ramming T, et al. Free Radic Biol Med. 2014, 70: 106-16.
ALDH1A1	Lei HM, et al. Theranostics. 2019, 9(24): 7122-7139.
CYP2E1	Lu Y, et al. Curr Pharm Des. 2018;24(14): 1502-1517.
FOXO3	Shang J, et al. Biochem Biophys Res Commun. 2020,533(4): 723-731.
FOXO1	Li K, et al. Stem Cell Res Ther. 2020, 11(1): 131.
MAFK	Zhang T,. et al. J Cell Physiol. 2019;234(3): 2511-2522.
MAFG	Zhang T, et al. J Cell Physiol. 2019, 234(3): 2511-2522.
TRADD	Kim JJ, et al. Cell Death Differ. 2010, 17(9): 1420-34.
SOX2	Liang Y, et al. Immun Inflamm Dis. 2020. doi: 10.1002/iid3.388.
ALCAM	Ishimoto T, et al.Cancer Cell. 2011, 19(3): 387-400.

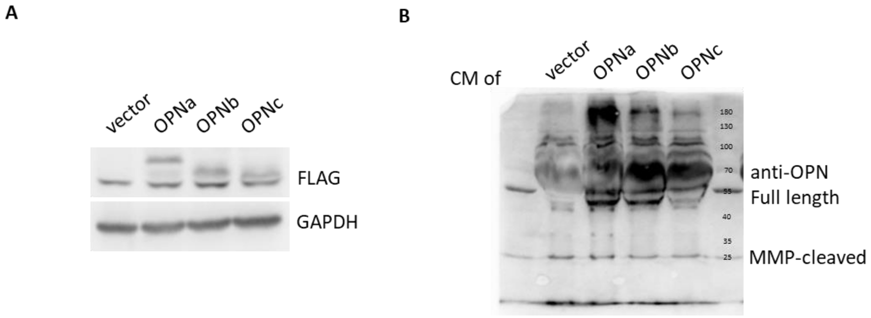

Suppl Figure 1. Western blot of OPN proteins in CMs from OPN-SIs overexpressing cells. **a** Cell lysate of transfected HEK-293T cells. **b** CMs collected from transfected HEK-293T cells.

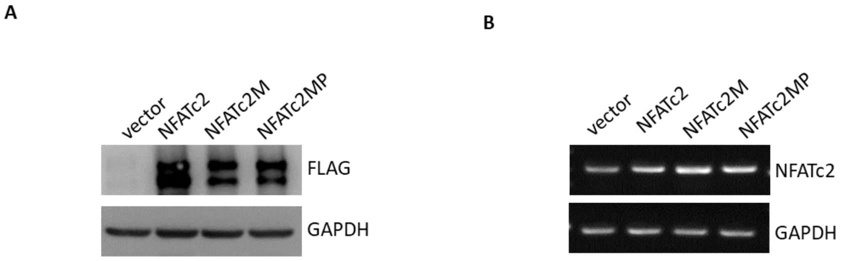

Suppl Figure 2. NFATc2 and its mutants were overexpressed in H1299 cells. **a** The protein levels of Flag-NFATc2 and its mutants were determined by western blotting in H1299 cells. **b** The mRNA levels of Flag-NFATc2 and its mutants were determined by RT-PCR in H1299 cells.

Suppl Figure 3 (Original images to Figure 3C)

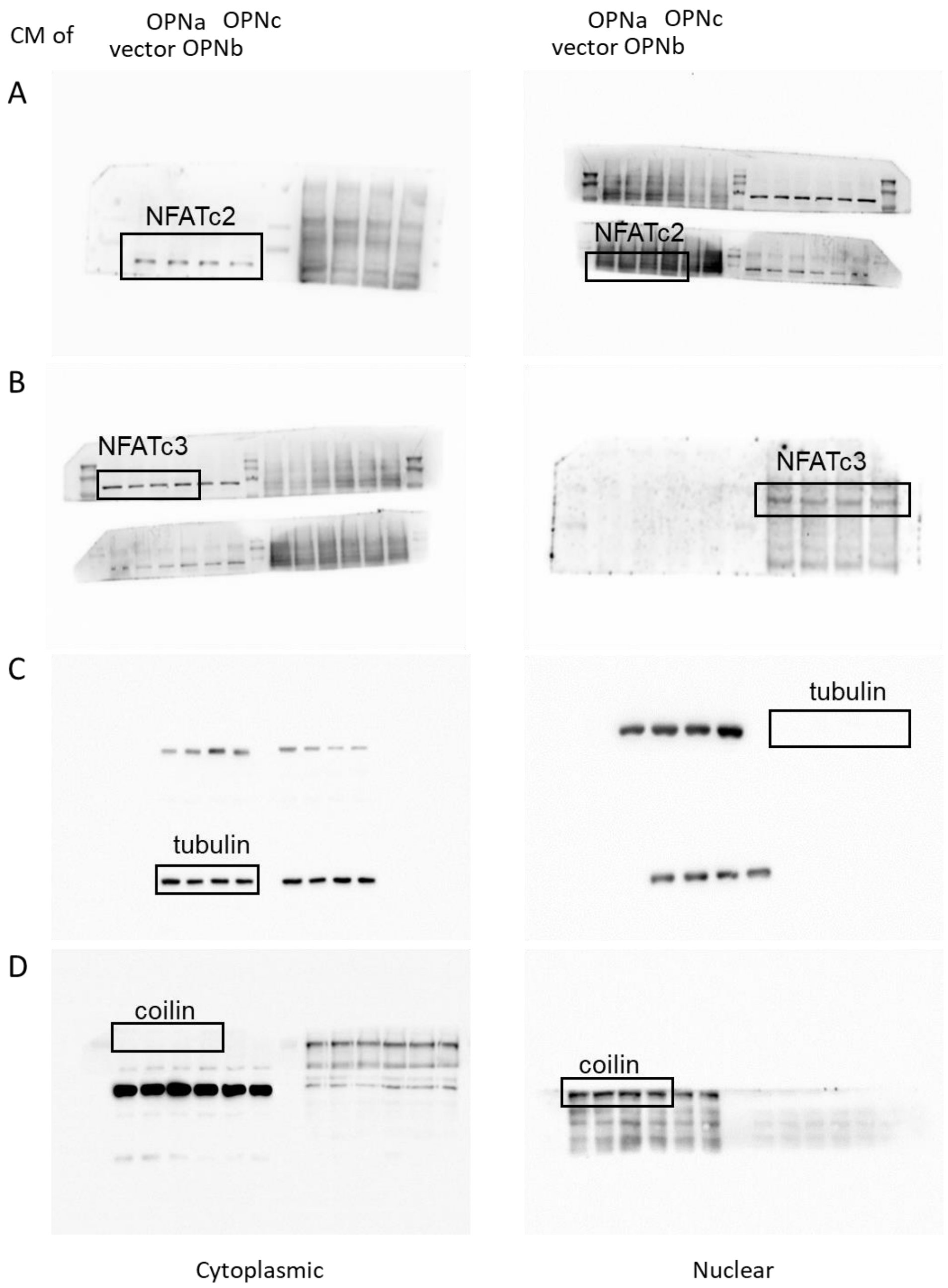

Suppl Figure 3 (Original images to Figure 3C). (**A**) Original image to Figure 3C-NFATc2. (**B**) Original image to Figure 3C-NFATc3. (**C**) Original image to Figure 3C-tubulin. (**D**) Original image to Figure 3C-coilin.

Suppl Figure 4 (Original images to Figure 3D)

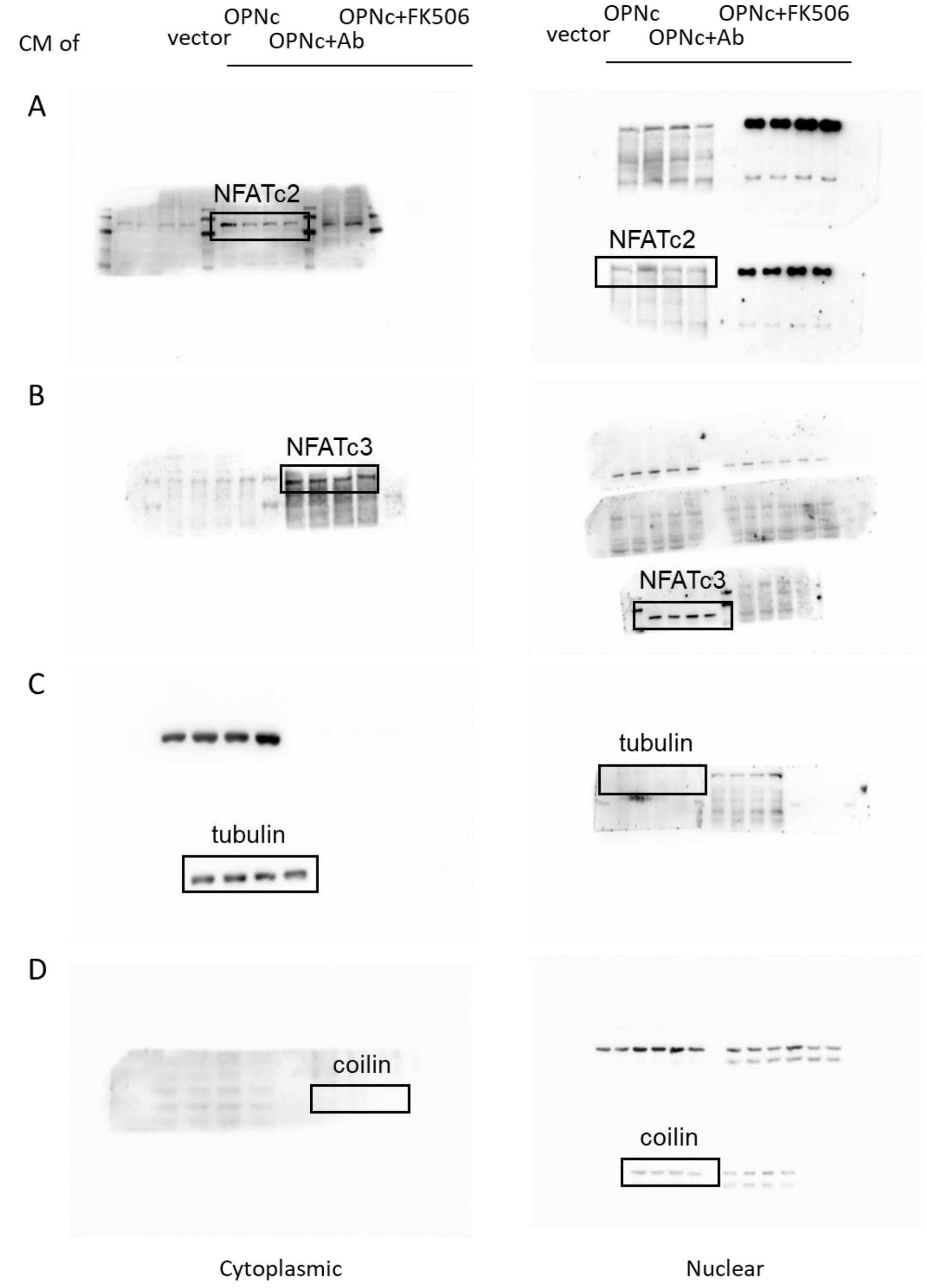

Suppl Figure 4 (Original images to Figure 3D). (**A**) Original image to Figure 3D-NFATc2. (**B**) Original image to Figure 3D-NFATc3. (**C**) Original image to Figure 3D-tubulin. (**D**) Original image to Figure 3D-coilin.

Suppl Figure 5 (Original images to Figure 6C)

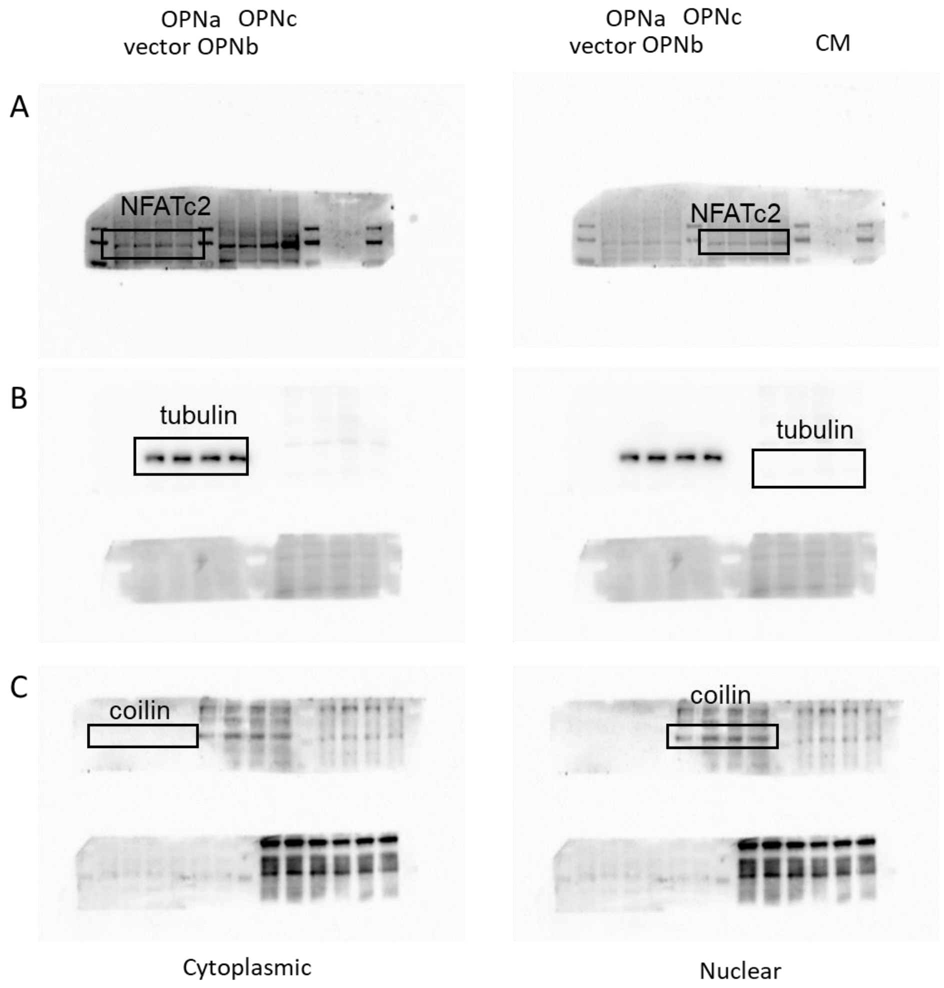

Suppl Figure 5 (Original images to Figure 6C). (**A**) Original image to Figure 6C-NFATc2. (**B**) Original image to Figure 6C-tubulin. (**C**) Original image to Figure 6C-coilin.

Suppl Figure 6

(Original images to Supplement Figure 1 and Supplement Figure 2)

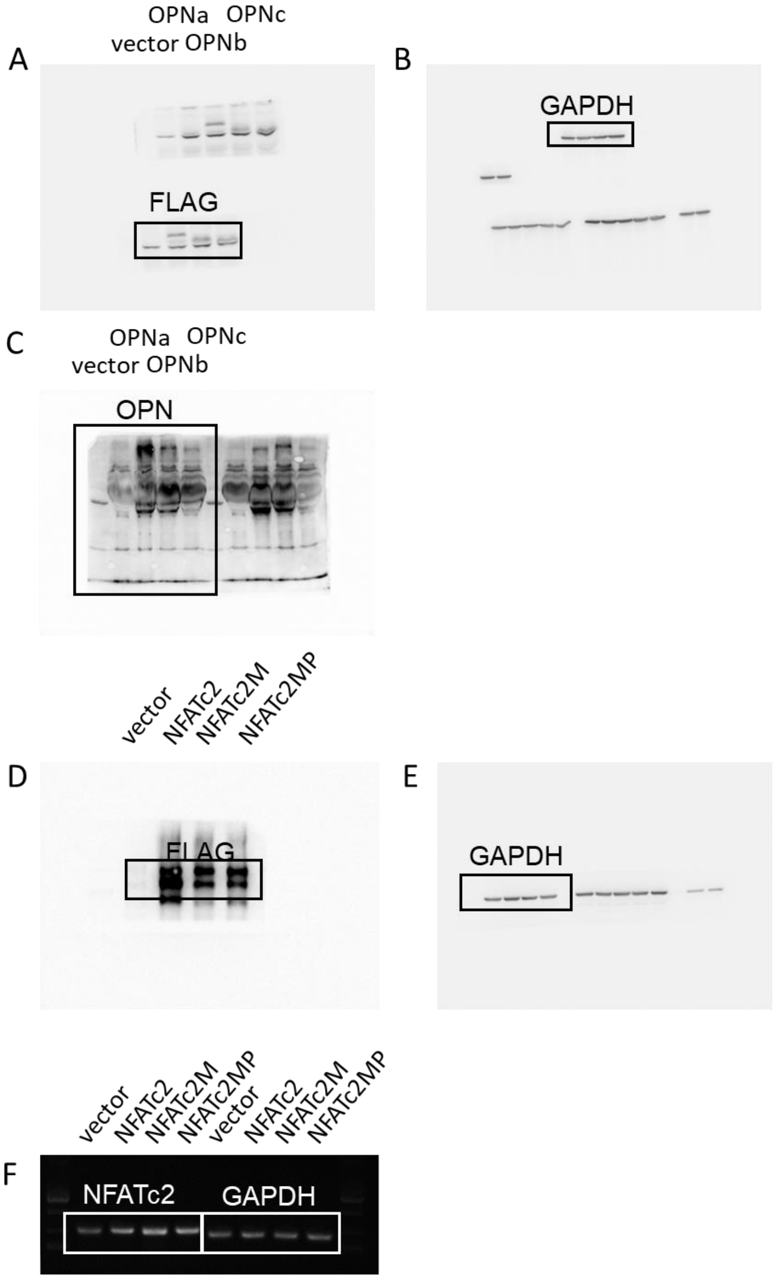

Suppl Figure 6 (Original images to Supplement Figure 1 and Supplement Figure 2). (**A**) Original image to Supplement Figure 1A-FLAG. (**B**) Original image to Supplement Figure 1A-GAPDH. (**C**) Original image to Supplement Figure 1B-OPN. (**D**) Original image to Supplement Figure 2A-FLAG. (**E**) Original image to Supplement Figure 2A-GAPDH. (**F**) Original image to Supplement Figure 2B-NFATc2 and GAPDH.
